# Buffalopox Virus: An Emerging Virus in Livestock and Humans

**DOI:** 10.3390/pathogens9090676

**Published:** 2020-08-20

**Authors:** Kamal H. Eltom, Abdallah M. Samy, Ahmed Abd El Wahed, Claus-Peter Czerny

**Affiliations:** 1 Unit of Animal Health and Safety of Animal Products, Institute for Studies and Promotion of Animal Exports, University of Khartoum, Shambat 13314, Khartoum North, Sudan; kamal@uofk.edu; 2 Entomology Department, Faculty of Science, Ain Shams University, Abbassia, Cairo 11566, Egypt; samy@sci.asu.edu.eg; 3 Division of Microbiology and Animal Hygiene, Georg-August University Göttingen, Burckhardtweg 2, D-37077 Göttingen, Germany; cczerny@gwdg.de; 4 Institute of Animal Hygiene and Veterinary Public Health, University of Leipzig, An den Tierkliniken 43, D-04103 Leipzig, Germany

**Keywords:** buffalopox, VACV, smallpox, orthopoxviruses

## Abstract

Buffalopox virus (BPXV) is the cause of buffalopox, which was recognized by the FAO/WHO Joint Expert Committee on Zoonosis as an important zoonotic disease. Buffalopox was first described in India, later in other countries, and has become an emerging contagious viral zoonotic disease infecting milkers with high morbidity among affected domestic buffalo and cattle. BPXV is a member of the genus *Orthopoxvirus* and a close variant of the vaccinia virus (VACV). Recent genome data show that BPXV shares a most recent common ancestor of VACV Lister strain, which had been used for inoculating buffalo calves to produce a Smallpox vaccine. Over time, VACV evolved into BPXV by establishing itself in buffaloes to be increasingly pathogenic to this host and to make infections in cattle and humans. Together with the current pandemic of SARS-COV2/COVID 19, BPXV infections illustrate how vulnerable the human population is to the emergence and re-emergence of viral pathogens from unsuspected sources. In view that majority of the world population are not vaccinated against smallpox and are most vulnerable in the event of its re-emergence, reviewing and understanding the biology of vaccinia-like viruses are necessary for developing a new generation of safer smallpox vaccines in the smallpox-free world.

## 1. Introduction

**Figure 1:**
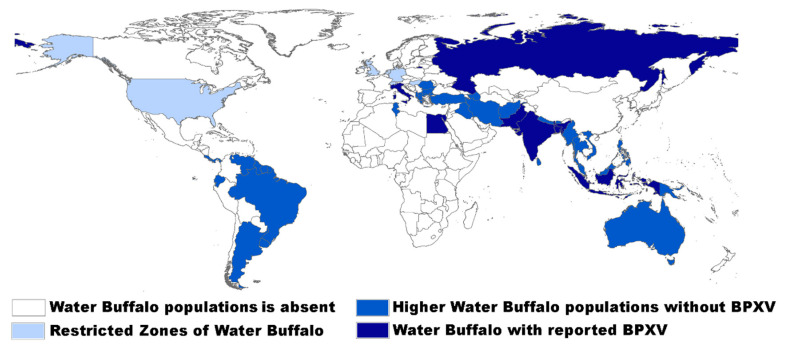
Water Buffalo and buffalopox virus distribution map.

Buffalopox virus (BPXV)—the etiological agent of buffalopox—is member of the genus *Orthopoxvirus*, subfamily *Chordopoxvirinae*, family *Poxviridae* https://talk.ictvonline.org/ictv-reports/ictv_9th_report/ [1]. BPXV is a close variant of the vaccinia virus (VACV), the type-species of the genus *Orthopoxvirus* (OPXV). Buffalopox was first described in India [2,3,4] and further reports on the disease came from other countries [5,6,7]. Discovery of the virus was achieved around the time of smallpox epidemics and the beginning of vaccination programs with VACV. The first isolation of the virus was made in northern India in the year 1967 and the virus continued to cause sporadic outbreaks in Asian buffaloes (*Bubalus bubalis*) in Bangladesh, India, Indonesia, Pakistan, Egypt, Russia, and Italy [8], Figure 1. In the same year of the first isolation of the virus, the disease was recognized by the FAO/WHO Joint Expert Committee on Zoonosis as an important zoonotic disease [9]. Forty years later, buffalopox became an emerging contagious viral zoonotic disease infecting milkers with high morbidity (80%) among affected domestic buffalo herds [10] and cows [11].

## 2. Virus Properties

Buffalopox virus resembles VACV in terms of its size, shape, structure, physico-chemical properties, and autonomous replication in “viroplasm zones” [6,12]. Buffalopox virus replicates in a wide range of cells [13,14,15,16,17]. Cell cultures of bovine and monkey origins are most frequently used for virus propagation, accompanied by a cytopathic effect (cpe). Whole-genome Restriction Fragment Length Polymorphism (RFLP) studies have indicated genetic similarity between VACV and BPXV [18]. Phylogenetic analysis based on some genes sequences [19,20] and complete genomes [21,22] revealed that BPXVs clustered closely with VACV rather than with other OPXVs. Furthermore, phylogenomics data support the hypothesis that VACV Lister and allied vaccine strains (Western Reserve, Copenhagen, etc.) share a most recent common ancestor with BPXV to the exclusion of other VACV strains [22]. Analysis of the available complete genomes of four isolates (three from India and one from Pakistan) confirmed the monophyly BPXVs [22].

## 3. Epidemiology, Host Range, and Immune Response

Epidemiology of buffalopox should be reconsidered more than 30 years after cessation of smallpox eradication campaigns [23]. BPXV resembles VACV in its pathogenesis, pathology, and histology. After recovery from infection, animals and humans are protected by both cell and antibody-mediated immunity. Neutralizing, hemagglutination-inhibiting, and precipitating antibodies are important for protection; they appear after 12 days approximately following experimental infection and maternal antibodies are transferred to the newborn animals via colostrum [6].

Many authors guessed that BPXV is likely to have emerged from the Lister vaccine strain of VACV, the strain that had been used in buffalo calves in India to produce smallpox vaccine [18,24,25]. Support for this has been provided by analyzing the complete genomes of BPXVs sequenced so far [21,22]. The emergence of BPXV occurred by gradual adaptation of the vaccine strain in buffaloes [26] until it converted to pathogenic, leading to outbreaks in this new host. Consequently, buffalopox outbreaks occurred frequently in many parts of India, affecting both buffaloes [10,17,27] and humans [10,28,29,30,31,32,33,34,35]. Reports on zoonotic outbreaks of buffalopox have been made from Pakistan as well [14,36]. Subsequently, reports on BPXV cases in other hosts, such as cattle, have also been made [11]. Transmission of the virus to cattle and humans is alarming as it might have serious public health implications, in the view that no more vaccination against smallpox is practiced since 1977 after its eradication [25]. The products of host-range genes have been demonstrated to affect the infecting ability of the virus for cells by subverting immune responses of the host [37]. The most important host-range genes of OPXVs that have been sequenced for BPXV strains are E3L, K3L, C7L, and B5R, which are implicated in altering the antiviral defense mechanism of the host cell. The full-length sequences of these four genes of BPXVs—obtained from outbreaks in buffaloes, cattle, and humans in India—were analyzed, to investigate their evolutionary relationship to other OPXVs circulating in the world vis-à-vis the vaccine strains. Sequences of these genes revealed a higher degree of similarity to those of VACV strains [26]. The functions of the VACV E3L gene were studied extensively in deletion mutants, which caused abortive replication and expression of only a subset of viral genes in most mammalian cell lines [38,39]. E3L encodes a 20-kDa and a 25-kDa protein that suppresses the antiviral response of the host cell by inhibiting both protein kinase and RNaseL [40,41]. The K3L gene confers interferon resistance and was shown to repress activation of the protein kinase (PKR) and phosphorylation of eIF2α in mammalian cells [42], which can result in inhibiting the antiviral defense mechanism. The C7L gene is conserved in all OPXV genomes [43] and causes inhibition of apoptosis [44] and blockage of antiviral effects by antagonizing interferons (IFN) [45]. The B5R gene is essential for the formation of extracellular virus particles (EV) [46] and is involved in viral evasion from the immune response of the host [47]. When this gene was deleted from VACV strain WR, it resulted in a decrease in EV production, reduction in the plaque size in vitro and in high attenuation of the virus in vivo in comparison with the parental strain [48]. Point mutation of at least one amino acid was observed within this gene in cattle and human isolates of BPXV [26], which also occurred when a BPXV isolate was passaged 50 times [14]. Some of these mutations might be critical for the virus to adapt to new hosts and can be implicated in the zoonotic nature of this virus [14].

## 4. Clinical Features

Clinical signs of buffalopox resemble those of VACV infections. Characteristic signs in buffaloes include a local pox exanthema (pustulation with central necrosis) and localized pock-lesions on the muzzle, udder, teats, inside of the thighs, scrotum, base of the ears, inner surface of earflap, and eyes in the mild form [6]. The disease may also proceed to severe systemic disease of a cyclical pattern with generalized lesions in individual cases [23].

Although buffalopox does not occur very frequently, the disease is economically important in countries where buffaloes are reared. The disease has a negative impact on the dairy industry as a consequence of reduced productivity (40–70% reduction) of affected milking animals when severe local pocks affect the udder and teats, which in turn may lead to mastitis [8,49].

Humans in close contact with affected animals can get infected by the virus. In humans, infection with BPXV was manifested as pox lesions in the flexor aspect of distal forearms, in the hand, dorsae of hands, wrist fingers, and thumbs, right preauricular area, right angle of mandible, right ala of nose, and forehead with or without swelling of the regional lymph nodes, general malaise, and fever [8,10,17,29,35,50].

Several reports on BXPV outbreaks involving both animals and humans or infections of individual cases were made from India. An outbreak of buffalopox in animals and humans in Maharashtra State of India in 2003 was reported. It involved 10 herds and resulted in 45% overall morbidity; some animals also exhibited lesions on their hindquarters, suggesting secondary or even a generalized infection. Milkers suffered pox-like local lesions on their hands, forearms, and forehead, presented with pyrexia, axillary lymphadenopathy, and general malaise [8,10]. Further similar outbreaks had also been described also in animals and humans [31,51] and a recent case report on human infection with BPXV was made on an Indian milkman and owner [29]. Manual milking with bare hands exposed these individuals to the infection.

A report on laboratory-acquired BPXV infection in humans was also made in India, highlighting the need for observance and enforcement of strict biosafety measures within laboratories [16].

In Pakistan in 2004–2005, reports were made describing a nosocomial outbreak of BPXV in humans in the five major burn units in Karachi. Here, patients developed pox lesions at burn wounds and the intact skin surrounding them. The source of infection was VACV-contaminated buffalo fat, which had been used as a first-aid medication for dressing the burns. This event showed an indirect mode of transmission of an OPXV [8,36].

## 5. Diagnosis

Although outbreaks of orthopoxvirus infections—unique to the Indian subcontinent region—were repeatedly described and significant veterinary research work was conducted in this respect, limited diagnostic tools were developed [15]. Clinical examination and collection of specimens (swab and serum) from both animals (buffaloes, cattle) and humans are the first steps of diagnosis. These samples are then subjected to electron microscopy examination, inoculation in cell culture for isolation of the virus, plaque reduction and neutralization test, PCR, and partial genome sequencing [15,49].

Like other OPXVs, BPXV can be isolated from the lesion scabs of animals and humans by inoculation in embryonated chicken eggs as well as in a number of cell lines including chick embryo fibroblast cells, pup kidney cells, Vero cells and baby hamster kidney cells [13,14,15,16,17]. Cytopathic effects can be observed in 3–4 days.

BPXV is serologically uniform and cross-reacts with both VACV and CPXV, as well as with other OPXVs. Therefore, serological assays are not advantageous for virus differentiation unless a targeted monoclonal antibody is used. Classical assays used to distinguish BPXV from other OPXVs would be double immunodiffusion (ID), complement fixation (CF), and immunoelectrophoresis (IE). Today, enzyme-linked immunosorbent assay (ELISA), immunofluorescence assays (IF), and Western blotting (WB), together with neutralization and plaque reduction tests (NT/PRT) are more commonly used. Earlier investigations with monoclonal antibodies have shown that BPXV is serologically more closely related to VACV than to other OPXVs [6,52]. Recent attempts were made for specific detection of BPXV and differentiation from other OPXVs. Monoclonal antibodies against the BP4 strain of BPXV were produced and used in antigen capture ELISA. Although these monoclonal antibodies differentiated the BPXV from other OPXVs, only two of them significantly bound different BPXV strains; none of them had virus-neutralizing abilities; furthermore, they did not bind the polypeptides shared by other BPXV strains in Western blotting [53]. Therefore, they are of no use for serodiagnosis of BPXV infections. Some recombinant proteins antigens were evaluated for the specific detection of BPXV [54,55]; they cross-reacted with other OPXV as they were based on conserved proteins (A27L and H3L) in OPXV. However, their potential use in diagnostic assays of BPX infections was not evaluated.

Primers for the C18L gene of OPXV were used in conventional PCR, duplex PCR, and real-time PCR [34] for the detection of BPXV. The primers amplify a 368 bp PCR product unique for BPXVs. In duplex PCR, using these primers together with those for the DNA polymerase (Pol DNA), OPXV species, as well as Capripox and Parapox viruses, amplified only a 96 bp amplicon of the Pol DNA, whereas BPXV amplified both the 368 bp and 96 pb PCR products. The sensitivity of real-time PCR, however, was 100 times more than the conventional PCR [34].

## 6. Therapy and Prophylaxis

Currently, no licensed specific antivirals are available for the treatment of BPXV infections in humans and animals. If possible, acute infections can be curtailed with immune sera; but, in the case of immunosuppressed individuals, serum therapy does not prevent the local infection from developing into a generalized systemic disease [23,56,57]. However, reservations against polyclonal human immune sera arise due to safety reasons, as their biological compounds are not characterized very well [23]. For the prevention of secondary bacterial infections, symptomatic treatment is provided. Because BPXV is closely related to VACV, the antivirals Cidofovir and ST246 may be effective for local and systemic treatment in humans and animals. Recently, a new series of thiazolo [3,2-a] pyrimidine-6-carboxylate derivatives 3a–f and 4a–f were synthesized and characterized [58]. The compounds were tested for in vitro antimicrobial and antiviral activities. The probable mode of action of these active compounds was determined through in silico docking study by docking the receptor methionyl-tRNA synthetase and human inosine-5′-monophosphate dehydrogenase (IMPDH) for antibacterial and antiviral activities, respectively. Of these compounds, 4c elicited excellent in vitro antimicrobial activity against all tested strains. On the other hand, compound 4a elicited 73.69% and 54.42% inhibition of the *Camelpox virus* (CMLV) and BPXV, respectively. Moreover, this compound exhibited minimum docking and binding energy along with the maximum hydrogen/hydrophobic interaction with IMPDH [58]. Recently, some protein kinase inhibitors were tested for antiviral activity. Among these, the kinase inhibitor CGP57380, which blocks the mitogen-activated protein kinase (MAPK) interacting kinase 1 (MNK1), was found to be promising as an antiviral agent against BPXV. In in vivo studies, this compound was found to decrease the synthesis of the viral genome and to reduce synthesis of viral proteins, whereas in *in ovo* studies it prevented the formation of pock lesions on the chorioallantoic membrane (CAM) as well as associated mortality of the chick embryos [59].

No specific vaccine against BPXV infection is available. However, prophylactic control and protection of animals in an infected herd is possible with a live vaccine based on an attenuated VACV strain. Vaccinia virus vaccines were initially produced in buffaloes (dermo-vaccine) in India [60]. Later cell culture adapted strains were used. The program resembles that of vaccinia and cowpox prophylaxis. However, the protective efficacy of the third generation vaccinia based vaccines has been tested in animals (mice, rabbit, and monkeys) [61]. In addition to the safety of these vaccines, the elicited immune responses provided protection against challenge with the respective virus. The use of these vaccines would be advantageous for use in buffalo and humans in contact with buffalo, as no data is available about prophylaxis in humans at risk of BPXV infections.

Recombinant DNA vaccines from the envelope proteins (A27L and 3HL) of OPXV—derived from a BPXV strain—were tested in animal models [54,55]. An increase in antigen-specific serum IgG level as well as in neutralizing antibody titers was observed in the recombinant vaccines. In passive protection experiments in suckling mice, hyperimmune sera of the recombinant A27L vaccine conferred 60% protection [54], while 80% protection was reached with anisera of the H3L [55]. A combined vaccine containing both A27L and H3L recombinant proteins elicited a high immune response in mice measure by specific IgG titers in ELISA and neutralizing antibody titers. In addition, complete protection of mice vaccinated with this combination was seen when they were challenged by virulent virus strain [62].

## 7. Future Aspects

Over time, BPXV evolved from VACV and established itself in buffaloes to be increasingly pathogenic to the new host, i.e., buffaloes, and further to make infections in cattle and humans [23]. The emergence of a pathogenic OPXV, which can spread efficiently from human-to-human, should be considered an immediate public health risk [63,64,65]. Together with the current pandemic of SARS-COV2/COVID 19, BPXV infections in India [10,17,28] illustrate how vulnerable the human population is to the emergence and re-emergence of viral pathogens from unsuspected sources. A deep understanding of the underlying molecular mechanisms, that control the species tropism of poxviruses in non-evolutionary hosts is of utmost importance [26]. As more information becomes available on tropism determinants of poxviruses, new strategies will likely be developed to control zoonotic infections. Raising awareness, improvement of diagnostic techniques, education, and preparedness for early intervention, and development of disaster guidelines are necessary given the potential disease outbreak, in case it happens in the future [15]. Like most OPXVs and other zoonotic poxviruses reservoir host(s) that maintain BPXV in the environment did receive attention and yet is unknown—although it is thought to be most likely rodents [8]; knowledge on this respect is essential for the prevention of introduction of the virus in naïve buffalo and cattle populations and for designing eradication and control programs.

Increasing incidences of OPXV infections are being reported across the world: BPXV in Asia, VACV and VACV-like viruses (VLVs) in Brazil [66], MPXV in East and Central Africa and the USA [67] and novel OXPVs (AKMV [68], AKPV [66], ECTV-like OPXV [69,70])—which are being described at an increasing rate. Emergence and re-emergence of these OPXVs are alarming in view that about 50% of the world population >30 years are not vaccinated against smallpox and are most vulnerable in the event re-emergence of this disease [33]. In addition, the rise in global bioterrorism necessitates the use of third generation smallpox vaccines, as they have been shown to be safer than preceding generations [61], in the most vulnerable populations. Furthermore, efforts should be increased for searching a new generation of safer smallpox vaccines, a necessary step towards which research should be directed for reviewing and understanding the biology of VLVs in the smallpox-free world.
